# Editorial Notes

**Published:** 1948

**Authors:** 


### Editorial Notes

**The Sheriff of Bristol.**

MR. HUBERT CHITTY, M.S., F.R.C.S., is the third ex-President of the Bristol Medico-Chirurgical Society to hold the office of Sheriff of Bristol and we are glad that he has the time and the inclination to undertake this responsibility. He has already shown with what dignity and energy he discharges his duties. His medical forerunners have been Mr. F. Richardson Cross who was Sheriff in 1898 and President of this Society in 1891–92, Dr. W. Kenneth Wills, Sheriff in 1932–33, President of the Society in 1927–28. Mr. Chitty accepted the Shrievalty in November last immediately after vacating the Presidential Chair, and retiring from active hospital work. Apparently, the Navy is a good training ground for Sheriffs of Bristol, for both Dr. Kenneth Wills and Mr. Chitty served as Navy Surgeons in the 1914–18 war.

**Swiney Prize for a work on Medical Jurisprudence.**

THE Council of the Royal Society of Arts give notice that the next award of the Swiney Prize will be made in January, 1949, the one-hundred-and-fifth anniversary of the testator's death. Dr. Swiney died in 1844, and in his will he left a sum of money to the Royal Society of Arts for the purpose of presenting a prize, on every fifth anniversary of his death, to the author of the best published work on Jurisprudence. The prize is a cup, in normal times of the value of £100, but on this occasion of the value of £150, and money to the value of £100.

The award is made by a joint Committee of the Royal Society of Arts and the Royal College of Physicians, which appoints special adjudicators.

The prize is offered alternately for Medical and General Jurisprudence, but if at any time the Committee is unable to find a work of sufficient merit in the class whose turn it is to receive the award, it is at liberty to recommend a book belonging to the other class. On the last occasion of the award (1944) the prize was awarded for General Jurisprudence. It will, therefore, be offered on the present occasion for Medical Jurisprudence.

Any person desiring to submit a work in competition, or to recommend any work for the consideration of the Judges, should send it to the Secretary of the Society, John Adam Street, Adelphi, London, W.C.2, not later than November 30th, 1948.

